# Calcium and Magnesium Ions Are Membrane-Active against Stationary-Phase *Staphylococcus aureus* with High Specificity

**DOI:** 10.1038/srep20628

**Published:** 2016-02-11

**Authors:** Yuntao Xie, Lihua Yang

**Affiliations:** 1 CAS Key Laboratory of Soft Matter Chemistry, University of Science and Technology of China, Hefei, Anhui 230026 China; 2 Department of Materials Science and Engineering, University of Science and Technology of China, Hefei, Anhui 230026 China; 3 Department of Polymer Science and Engineering, University of Science and Technology of China, Hefei, Anhui 230026 China

## Abstract

*Staphylococcus aureus* (*S. aureus*) is notorious for its ability to acquire antibiotic-resistance, and antibiotic-resistant *S. aureus* has become a wide-spread cause of high mortality rate. Novel antimicrobials capable of eradicating *S. aureus* cells including antibiotic-resistant ones are thus highly desired. Membrane-active bactericides and species-specific antimicrobials are two promising sources of novel anti-infective agents for fighting against bacterial antibiotic-resistance. We herein show that Ca^2+^ and Mg^2+^, two alkaline-earth-metal ions physiologically essential for diverse living organisms, both disrupt model *S. aureus* membranes and kill stationary-phase *S. aureus* cells, indicative of membrane-activity. In contrast to *S. aureus*, *Escherichia coli* and *Bacillus subtilis* exhibit unaffected survival after similar treatment with these two cations, indicative of species-specific activity against *S. aureus*. Moreover, neither Ca^2+^ nor Mg^2+^ lyses mouse red blood cells, indicative of hemo-compatibility. This works suggests that Ca^2+^ and Mg^2+^ may have implications in targeted eradication of *S. aureus* pathogen including the antibiotic-resistant ones.

*Staphylococcus aureus* (*S. aureus*) is a Gram-positive bacterium notorious for its ability to acquire antibiotic-resistance^1^. Antibiotic-resistant strains of *S. aureus* have emerged as a widespread cause of both hospital- and community-associated infections, leading to high mortality rate^1^,^2^,^3^. For example, methicillin-resistant *S. aureus* is estimated to cause >11,000 deaths per year in the United States^4^. It is thus imperative to discover/develop antimicrobials that are both active against *S. aureus* including antibiotic-resistant strains and less prone to evoke resistance.

Antimicrobial peptides (AMPs) are nature’s antibiotics still in action despite of their evolutionarily ancient origins. Many AMPs act by impairing the barrier function of bacterial membranes^5^,^6^,^7^,^8^,^9^,^10^,^11^, a generic mode that appears to be more difficult for bacteria to circumvent than the metabolic-targeting modes of conventional antibiotics^12^. By capturing the structural features common to most AMPs (*i.e.*, being simultaneously cationic and amphiphilic), synthetic mimics of AMPs (SMAMPs) have demonstrated similar *in vitro* antibacterial activity and membrane-destabilizing modes as do AMPs^13^,^14^,^15^,^16^,^17^,^18^,^19^,^20^,^21^,^22^,^23^,^24^,^25^,^26^,^27^,^28^,^29^,^30^,^31^,^32^,^33^,^34^,^35^,^36^,^37^,^38^,^39^,^40^,^41^,^42^. Despite of the great potentials, AMPs and SMAMPs are effort- and, often, cost-consuming to produce, which has significantly hindered their pharmaceutical development. Alternative to AMPs and SMAMPs, species-specific antimicrobials have recently been proposed as a promising source of anti-infective agents that are less prone to evoke resistance^43^. Therefore, antimicrobials that are readily available and simultaneously membrane-active and species-specific against *S. aureus* are highly desired.

Metal ions are readily available, and certain transition-metal ions (*e.g.*, Cu^2+^, Hg^2+^, Zn^2+^, and Cd^2+^) have demonstrated wide-spectrum antibacterial efficacy to varying extent^44^,^45^. A best known example might be Ag^+^ ion, which is active against both Gram-negative and –positive bacteria^46^,^47^. Nevertheless, use of heavy metal ions as disinfectants may adversely impact the host and/or the environment. Besides, the action modes by which these heavy metal ions achieve their antibacterial activity remain elusive. Having these concerns in mind, we hence turn to non-transition metal ions, in efforts to find candidates for specifically disrupting *S. aureus* membranes.

Calcium and magnesium ions (Ca^2+^ and Mg^2+^) are two alkaline-earth-metal ions (M^2+^) physiologically essential to almost all living organisms^48^. Upon binding with cardiolipin (CL), a major lipid component in *S. aureus* membranes^49^,^50^, M^2+^ (M = Ca, Mg) forms M^2+^-CL complexes of negative curvature (Fig. 1a)^41^,^51^, and negative curvature is a physical parameter necessary for a variety of membrane-destabilization processes as has been validated for those induced by AMPs and SMAMPs^11^,^39^,^40^,^41^,^42^. We therefore hypothesize that Ca^2+^ and Mg^2+^ may be membrane-active, species-specific agent against *S. aureus*. To test this hypothesis, we perform vesicle membrane permeabilization assays and antibacterial assays and find that, at ≤40 mM, both Ca^2+^ and Mg^2+^ disrupt model *S. aureus* membranes and kill stationary phase *S. aureus* cells, indicative of membrane-activity. In contrast to *S. aureus*, *Escherichia coli* and *Bacillus subtilis* exhibit unaffected survival after similar treatment with these two cations, indicative of species-specific activity against *S. aureus*. Moreover, within the tested dose range, neither Ca^2+^ nor Mg^2+^ is hemolytic against mouse red blood cells, indicative of good hemo-compatibility. Collectively, these results suggest that Ca^2+^ and Mg^2+^ may have implications in targeted eradication of *S. aureus* pathogen including antibiotic-resistant ones.

## Results and Discussion

**Figure 1:**
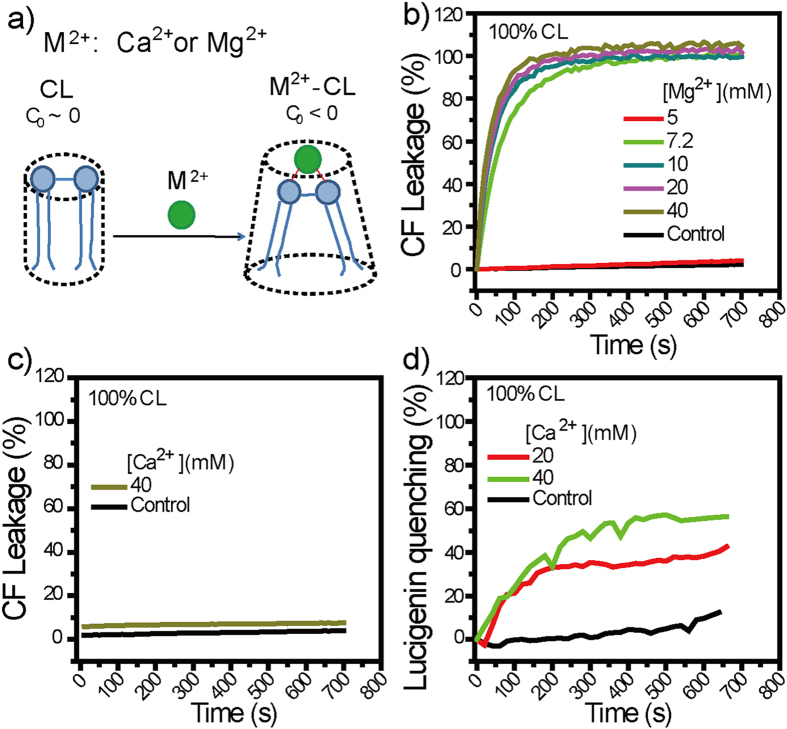
(**a**) Binding of M^2+^ (M = Ca, Mg) with cardiolipin (CL), the major lipid component in *S. aureus*, converts the originally zero-intrinsic-curvature (*C*_0 _~ 0) lipid into M^2+^-CL complexes with negative intrinsic curvature (*C*_0_ < 0). (**b–d**) Dye leakage assays using large unilamellar vesicles (LUVs) composed of 100% CL as a first-order model for *S. aureus* membranes. (**b**) Mg^2+^ at ≥7.2 mM caused significant carboxyl fluorescein (CF) leakage. (**c,d**) Ca^2+^, though (**c**) unable to cause detectable CF leakage, caused (**d**) appreciable quenching in the fluorescence intensity of intravesicular lucigenin. Controls are samples assayed similarly but without M^2+^ additions.

M^2+^ (M = Ca, Mg) binds with CL to form M^2+^-CL complexes of negative curvature (Fig. 1a)^41^,^51^, and negative curvature promote membrane destabilization as validated for cases with AMPs and SMAMPs^11^,^39^,^40^,^41^,^42^. We hence evaluated whether Ca^2+^ and Mg^2+^ destabilizes *S. aureus* membranes, using mono-component large unilamellar vesicles (LUVs) composed of CL as our first order model of *S. aureus* membranes and performing dye leakage assays^39^,^41^,^52^,^53^,^54^,^55^,^56^,^57^. To dissect the effect of M^2+^ dose from those of ionic strength and osmolarity, we use M^2+^-supplemented HEPES buffers (Table 1) which have ionic strength and osmolarity kept almost constant but varying MCl_2_ concentration; these same buffers are used for all experiments throughout this work. The first dye probe we used is carboxyl fluorescein (CF), a membrane-impermeant molecule with a hydrodynamic diameter of ~1 nm and negatively charges at physiological pH^58^,^59^,^60^,^61^,^62^. Mg^2+^, once ≥7.2 mM, caused ~100% CF leakage from CL LUVs (Fig. 1b), indicative of Mg^2+^-induced CF efflux across CL membranes. Ca^2+^, though unable to cause appreciable CF leakage up to 40 mM (Fig. 1c), induced >30% lucigenin quenching (Fig. 1d) once ≥20 mM in similar assays but with CF being replaced with lucigenin—a membrane-impermeant, fluorescent Cl^−^-indicator of similar molecular diameter as CF^63^,^64^,^65^, indicative of Ca^2+^-induced Cl^−^ influx across membranes. Obviously, both Ca^2+^ and Mg^2+^ permeabilize CL membranes. That their distinct abilities to releases CF from CL LUVs correlate with the difference in water channel diameter of inverted hexagonal (*H*_*II*_) structures they induced in CL membranes (1.50 nm *versus* 2.42 nm)^51^ further suggest that they may cause the observed membrane permeabilization by binding with CL to form negative-curvature M^2+^-CL complexes (M = Ca, Mg).

**Figure 2:**
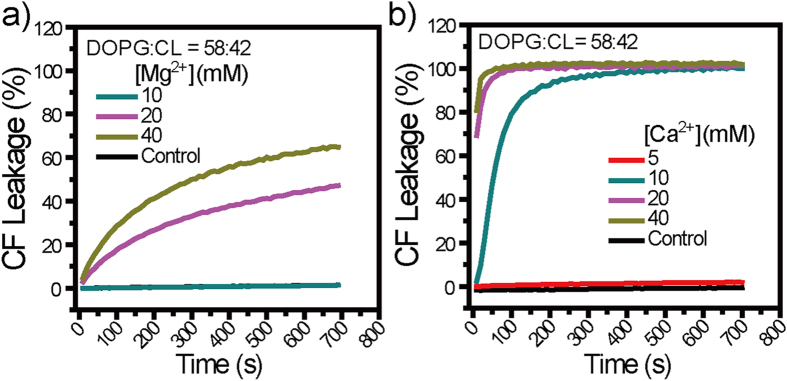
(**a,b**) Dye leakage assays using DOPG: CL = 58:42 LUVs as a more realistic model for *S. aureus* membranes. Obviously, both (**a**) Mg^2+^ and (**b**) Ca^2+^ caused significant CF leakage from DOPG: CL = 58:42 LUVs and, to do so, their minimum threshold concentrations are 20 and 10 mM, respectively. Controls are samples assayed similarly but without M^2+^ addition.

In addition to CL, phosphoglycerol (PG) is another major lipid component in *S. aureus* membranes^49^,^50^. To assess whether Ca^2+^ and Mg^2+^ permeabilize *S. aureus* membranes in which CL is diluted by PG, we use binary LUVs composed of DOPG:CL = 58:42 as a more realistic model for *S. aureus* membranes^57^ and perform similar CF leakage assays as above. From DOPG:CL = 58:42 LUVs, Ca^2+^ at ≥10 mM caused >90% CF leakage within 300 s after its addition, as compared to undetectable CF leakage caused by that at 5 mM (Fig. 2a), indicative of a minimum threshold Ca^2+^ concentration of 10 mM. Similarly, Mg^2+^ at ≥20 mM caused ≥40% CF leakage at 700 s after its addition, as compared to undetectable CF leakage by that at ≤10 mM (Fig. 2b), indicative of a minimum threshold Mg^2+^ concentration of 20 mM. Obviously, both Ca^2+^ and Mg^2+^ permeabilize model *S. aureus* membranes despite that CL content is diluted by PG but, for them to do so, certain minimal threshold concentrations are required.

**Figure 3:**
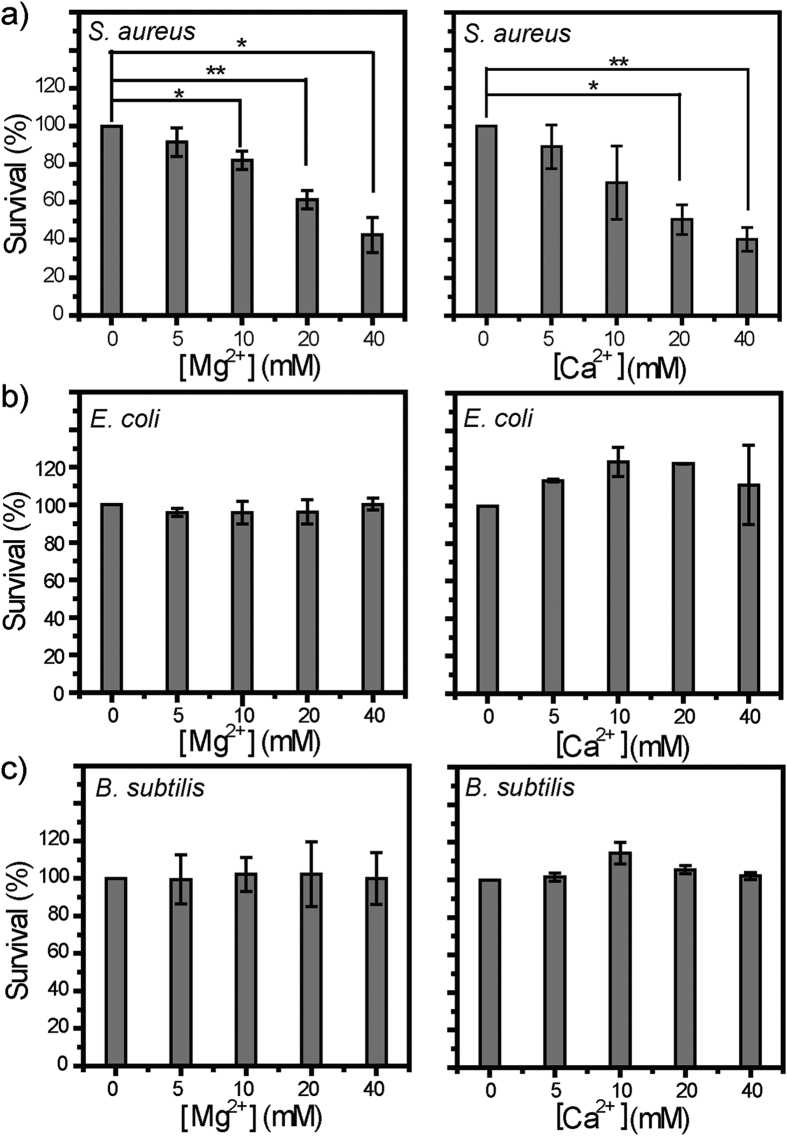
Plate killing assays against stationary-phase cells of (**a**) *S. aureus*, (**b**) *E. coli*, and (**c**) *B. subtilis*. After 40-min co-incubation with Mg^2+^ (left) and Ca^2+^ (right) at ≤40 mM, *S. aureus* cells exhibited dose-dependent loss in viability. In contrast, the viability of *E. coli* and *B. subtilis* cells is barely impacted in similar assays. Data points are reported as mean ± standard deviation. *and** indicate *p* < 0.05 and *p* < 0.01, respectively.

Both Ca^2+^ and Mg^2+^ are active against model *S. aureus* membranes. Does that necessarily correspond to antibacterial activity against *S. aureus* cells? To assess this, we evaluated the bactericidal activities of Ca^2+^ and Mg^2+^ by performing antibacterial plate killing assays. Note that bacterial cells in stationary phase are more resistant to environmental stresses and antibiotics than counterparts in logarithmic phase^12^,^66^,^67^. We hence used *S. aureus* cells in stationary phase, rather than those in logarithmic phase as normally do, for antibacterial assays. Our results (Fig. 3a) reveal that, after 40-min treatment with either Ca^2+^ or Mg^2+^, *S. aureus* cells exhibit viability loss to varying extent in a dose-dependent manner, with a maximal viability loss of ~60% observed at M^2+^ concentration of 40 mM, the highest dose tested. It is noteworthy that a relative loss of 60% in viability ratio corresponds to an absolute number density of ~3 × 10^5^ CFU/mL (colony-forming units per milliliter) in bacterial cells killed. Taken together, these observations suggest that both Ca^2+^ and Mg^2+^ are definitively bactericidal against *S. aureus*.

Closer examinations on both membrane permeabilizaiton experiments and antibacterial assays above suggest that Ca^2+^ and Mg^2+^ may be membrane-active against *S. aureus*. The minimal M^2+^ dose required for killing significant (*p* < 0.05) percentage of inoculated *S. aureus* cells are 10 and 20 mM for Ca^2+^ and Mg^2+^, respectively (Fig. 3a), which correlate well with the respective minimal threshold M^2+^ dose for these two cations to induce appreciable CF release from model *S. aureus* membranes (Fig. 2a,b), suggesting that Ca^2+^ and Mg^2+^ may kill *S. aureus* cells by disrupting their membranes.

In stark contrast to their definitive activity against *S. aureus*, Ca^2+^ and Mg^2+^ barely affect the viability of *E. coli* or *B. subtilis* (Fig. 3b,c), despite that *B. subtilis* is a Gram-positive bacterium as is *S. aureus*. Both *E. coli* and *B. subtilis* contain no/low CL in their membranes^68^. Thus, high CL content in *S. aureus* membrane may account for the observed activity of Ca^2+^ and Mg^2+^.

With species-specific antibacterial activity, MCl_2_ solutions are distinct from their corresponding MO powder slurries, which are wide-spectrum disinfectants^69^,^70^,^71^,^72^,^73^ used by human population of different cultures. Moreover, the observed activity of MCl_2_ solutions suggests that M^2+^ (M = Ca, Mg) ions may play contributive, rather than negligible, roles in the activity of their corresponding metal oxide (MO) powder slurries against *S. aureus*. To inhibit/kill ≥50% inoculated cells requires MO powder slurries of MO power dose at a few mg/mL, which corresponds to 1–100 mM^70^,^71^,^74^,^75^. Frequently, M^2+^ (M = Ca, Mg) ions produced *via* MO dissociation are viewed as negligible factors in the antibacterial activities of MO powder slurries^74^,^76^,^77^, due to inactivity of both the supernatant of MgO powder slurry and the MCl_2_ solutions at concentrations 10-fold of the MO powder solubility values^70^,^72^. Note that M^2+^ concentrations even 10-fold of MO powder solubility values are still <10 mM, which is within the barely-active dose range (Fig. 3a). Moreover, presence of *S. aureus* cells may actively retrieve free M^2+^ to form M^2+^-CL complexes, a process which may promote MO dissociation and thus shift the effective M^2+^ concentrations into the bactericidal range.

**Figure 4:**
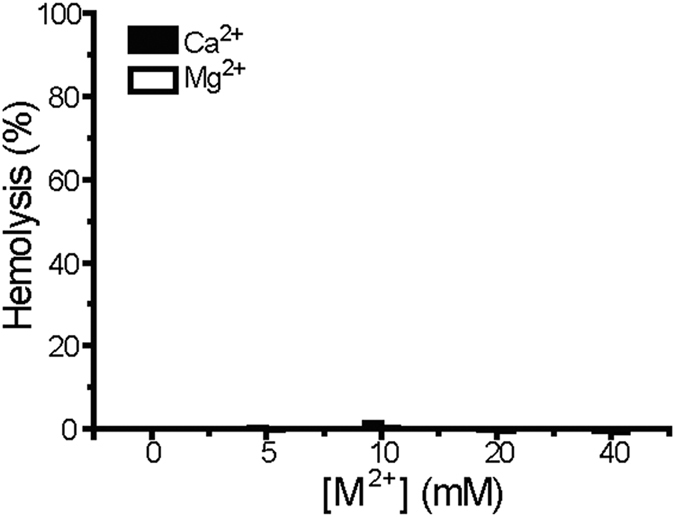
Up to 40 mM, neither Mg^2+^ nor Ca^2+^ caused detectable hemolysis against mouse red blood cells. Data points are reported as mean ± standard deviation.

As potential antimicrobial agents, toxicity to host cells is a major concern. To preliminarily evaluate the toxicity of Mg^2+^ and Ca^2+^ ions, we performed hemolytic assays against mouse red blood cells. Within the tested concentration range (0–40 mM), neither Mg^2+^ nor Ca^2+^ caused >5% hemolysis (Fig. 4), indicative of good hemo-compatibility. Combined with the antibacterial assays (Fig. 3), these results suggest that Mg^2+^ and Ca^2+^ may preferentially eradiate *S. aureus* cells without affecting other bacteria or mammalian cells in the same niche.

## Conclusion

In summary, we found that Ca^2+^ and Mg^2+^ may be membrane-active, species-specific bactericidal agent against *S. aureus*. Moreover, within the tested concentration range, both Ca^2+^ and Mg^2+^ lack hemolytic toxicity. This work suggests that Ca^2+^ and Mg^2+^ may have implications in targeted eradication of *S. aureus* pathogen including antibiotic-resistant ones.

## Methods

### Materials

*S. aureus* (ATCC 25923), *E. coli* (ATCC 25922) and *B. subtilis* (ATCC 6051) were purchased from American Type Culture Collection (ATCC) (Virginia, USA). Lipids used in this work, DOPG (1,2-dioleoyl-snglycero-3-[phospho-rac-(1-glycerol)] (sodium salt)), and TOCL (1,1′,2,2′-Tetraoleoyl Cardiolipin, Sodium Salt) were purchased from Avanti Polar Lipids (Alabama, USA) and used without further purification. Carboxyl fluorescein (CF) was purchased from Sigmal-Aldrich (Shanghai, China). Dehydrated Mueller-Hinton (MH) medium formulation and dehydrated Tryptic Soy Broth (TSB) medium formulation were purchased from Qingdao Hope Bio-Technology (Qingdao, China). All other reagents were purchased from Sinopharm Chemical Reagent Company (Shanghai, China). All reagents were used as supplied unless specified otherwise.

### Large Unilamellar Vesicle (LUV) Preparations

LUVs composed of 100% CL and DOPG:CL = 58:42 were used as model cellular membranes for *S. aureus*^57^ and prepared via extrusion. Into a glass vial, CL stock solution was added with or without stock solutions of DOPG; all lipid stock solutions were in chloroform at 20 mg/mL. The resulting lipid mixture was dried under gentle N_2_ flow, desiccated in vacuum overnight, and rehydrated with CF (40 mM CF) or lucigenin (1 mM lucigenin, 50 mM NaNO_3_) solutions at 45 °C for 2 h. The resultant solution was subjected to five freeze-thaw cycles and subsequently extruded through a 0.4-*μ*m Nucleopore polycarbonate membrane (Whatman) for 21 times using a mini-extruder (Avanti Polar Lipids). External CF or lucigenin was removed by gel filtration (Sephadex G-25, GE healthcare) using HEPES buffer A (10 mM HEPES, 150 mM NaCl, pH = 7.4) as eluent.

### Characterizations on Membrane-Permeabilization

Fluorescence emission intensity *I*_t_ for CF (λ_ex_ = 492 nm, λ_em_ = 518 nm) was monitored as a function of time (*t*). Into a fluorimeter sample cuvette, we added expected CF-preloaded LUV suspension (900 *μ*L) and, at 200 s after initiation of *I*_t_ recording, M^2+^-containing HEPES solutions (1800 *μ*L in total, Table 1), to a final lipid concentration of 0.1 mM. At 900 s after initiation of *I*_t_ recording (*i.e.*, at 700 s after M^2+^ addition), 50 *μ*L 10% triton was added, to yield *I*_∞_ which indicates 100% CF leakage. The percentages of CF leakage were calculated as leakage (%) = (*I*_t_ − *I*_0_)/(*I*_∞_ − *I*_0_) × 100, where *I*_0_ is the fluorescence intensity immediately (<10 s) after M^2+^ addition. Controls are samples assayed similarly but treated with M^2+^-absent solution (*i.e.*, 10 mM HEPES, 150 mM NaCl, pH 7.4).

### Antibacterial Assays

The antibacterial activities of Ca^2+^ and Mg^2+^ were evaluated by performing classic plate killing assays against stationary-phase bacterial cells. For each bacterial strain, 3–5 individual bacterial colonies were inoculated into fresh sterile trypticase soy broth (TSB) and incubated at 37 °C with shaking (200 rpm) for 16 h to stationary phase. Bacterial cells were then harvested and washed twice with sterile HEPES buffer A (10 mM HEPES, 150 mM NaCl, pH 7.4) *via* centrifugation (2,500 rpm, Eppendorf 5810R) for 1 min and, within 15 min, adjusted with sterile HEPES buffer A (10 mM HEPES, 150 mM NaCl, pH 7.4) to ~1.5 × 10^6 ^CFU/mL and inoculated (50 *μ*L) into each zero-dilution well (150 *μ*L in total) of a preset 96-well microplate.

Expected amounts of divalent cation stock solutions (10 mM HEPES, 150 mM CaCl_2_ or MgCl_2_, pH 7.4), sucrose stock solution (10 mM HEPES, 400 mM sucrose, pH 7.4), NaCl stock solution (10 mM HEPES, 500 mM NaCl, pH 7.4), and HEPES buffer B (10 mM HEPES, pH 7.4) were added into each zero-dilution well of a 96-well plate; all solutions were sterilized *via* filtering. After bacterial inoculation, final inculum size in each zero-dilution well was ~5 × 10^5 ^CFU/mL and final buffer compositions in the zero-dilution wells are summarized in Table 1.

The microplate was then incubated at 37 °C with shaking (200 rpm) for 40 min. Serial 10-fold dilutions were subsequently made with sterile HEPES buffer A (10 mM HEPES, 150 mM NaCl, pH 7.4). Each dilution (20 *μ*L) was plated onto MH agar plates, which were then incubated at 37 °C overnight to give visible colonies. Inoculum size was indicated by control samples containing untreated bacteria. Each trial was performed in triplicate, and the reported results are the averages of two independent trials.

### Hemolysis assays

Mouse blood was withdrawn from healthy mice obtained from the Animal Center of Anhui Medical University; the animal treatment was performed in compliance with the guidelines for the care and use of research animals established by the Animal Care and Use Committee at University of Science and Technology of China, and the experimental protocol was approved by the Animal Care and Use Committee at University of Science and Technology of China. Fresh mouse blood (200 μL) was washed with sterile HEPES buffer (10 mM HEPES, 150 mM NaCl, pH = 7.4) (800 μL) and washed for three times with sterile HEPES buffer via centrifuge at 900 rcf for 5 min, and the pellet was re-suspended into sterile HEPES buffer (1,000 μL) to yield the mouse red blood cell (mRBC) stock suspension for hemolysis assays. The mRBC stock suspension (200 μL) and MCl_2_-containing HEPES solution (400 μL) were added into each centrifuge cups. After the incubation at 37 °C for 40 min with shaking at 250 rpm, the centrifuge cups were centrifuged at 900 rcf for 5 min, and the supernatant (100 μL) of each cup was transferred into a well of a 96-well microplate. Hemolysis was monitored by measuring the absorbance of the released hemoglobin at optical density at 414 nm, OD_414_. Controls included HEPES buffer (300 μL) and mRBC suspension (200 μL) treated with triton X-100 (50%, 100 μL) to provide reference for 0% and 100% hemolysis, respectively. Each hemolysis assay trial was carried out in triplicate, and the reported results are the averages of two independent trials.

### Statistical Analysis

Statistical comparisons were carried out by performing student t test analysis with the statistical software package BioMedCalc (version 2.9). *p* values of <0.05 and <0.01 indicate statistical difference and statistically significant difference, respectively.

## Additional Information

**How to cite this article**: Xie, Y. and Yang, L. Calcium and Magnesium Ions Are Membrane-Active against Stationary-Phase *Staphylococcus aureus* with High Specificity. *Sci. Rep.* **6**, 20628; doi: 10.1038/srep20628 (2016).

## Figures and Tables

**Table 1:** Mg^2+^ and Ca^2+^ doses in 10 mM HEPES buffer^a^.

M^2+^ (mM)	HEPES (mM)	NaCl (mM)	Sucrose (mM)
0	10	170	0
5	10	155	0
10	10	140	15
20	10	110	45
40	10	50	105

^a^HEPES, NaCl, and sucrose were supplemented to help keep the pH, final ionic strength and final osmolarity constant at 7.4, 170 mM, and 325–340 RT (where R is the gas constant and T is ambient temperature, and ideal solutions are assumed), respectively. Same buffers were used for all experiments through this work unless specified otherwise.
